# Supplementary figures and images for: The Paf1 complex positively regulates enhancer activity in mouse embryonic stem cells
Source: Life Sci Alliance. 2020 Dec 29;4(3):e202000792. doi: 10.26508/lsa.202000792 (PMC7772781; doi:10.26508/lsa.202000792)

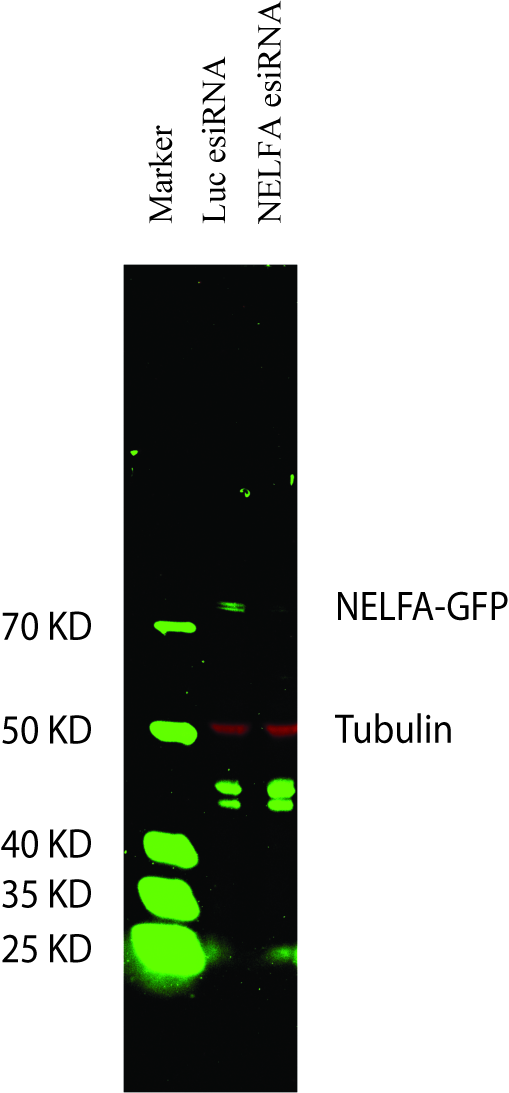

Supplement: Supplementary file 3 [file LSA-2020-00792_SdataFS3.tif]
